# Metagenomic sequencing reveals the taxonomic and functional characteristics of rumen microorganisms in Dongliu buffalo

**DOI:** 10.1038/s41598-025-03059-8

**Published:** 2025-05-26

**Authors:** Wenwen Lu, Jinling Hua, Min Zhang, Longfei Yan, Huwei Zhao, Xiaokang Lv

**Affiliations:** College of Animal Science, Anhui Science and Technology University, Fengyang, China

**Keywords:** Rumen, Metagenome, Carbohydrate degradation, Fiber degradation mode, Microbiome, Zoology

## Abstract

In this study, the composition of the rumen microbiota and its functional characteristics were investigated using a metagenomic approach in Dongliu buffalo. This study compared the rumen microbial communities of six female and four male Dongliu buffaloes of similar age, weight and lifestyle. Taxonomic analysis identified 964 genera across 52 phyla, dominated by *Bacteroidota* (47.54%) and *Bacillota* (28.20%). While alpha and beta diversity showed no sex differences (PERMANOVA *P* = 0.82), males exhibited higher *Fibrobacter* at the genus level (*P* = 0.02). Functional profiling revealed 429 KEGG pathways, with carbohydrate metabolism (11.17%) and amino acid metabolism (9.74%) as dominant processes. Males showed enrichment in cellulose-degrading enzymes (EC2.4.1.20, EC1.2.1.90, EC2.7.1.58) and CAZymes (GH94, GT35), while females had higher Bacteroides abundance (*P* = 0.01) and CAZymes like CBM47. Core cellulolytic genera (*Prevotella, Ruminococcus*) demonstrated male-biased GH/CBM activity, linked to enhanced fiber degradation. COG annotation highlighted carbohydrate metabolism as central, with sex-specific functional partitioning in replication (female-enriched) and secondary metabolism (male-enriched). Network analysis revealed *Prevotella’s* dominance in CAZymeme contributions and functional specialization in lignocellulose degradation pathways, suggesting sex-driven microbial adaptation to dietary fiber utilization.

## Introduction

The Dongliu Buffalo, often referred to as the swamp buffalo, is predominantly bred for service and meat production in the riverside and lake regions of Anhui, China^1^. Recognised as an exceptional swamp buffalo breed, it thrives on a diet of straw, reeds, pampa sticks and other vegetation, and is esteemed for the superior quality, color, and flavor of its meat. In contrast to other ruminants, buffaloes have a greater efficiency in utilizing roughage of lower quality, along with agricultural and industrial by-products rich in lignocellulosic substances^2,3^. This capability allows them to adjust to the crop residues available in their environment, resulting in the production of high-quality animal products, including meat and milk^4,5^. The rumen, a fermentative ecosystem adept at fiber lysis, hosts a diverse microbial community consisting of bacteria, protozoa, archaea, fungi, and bacteriophages. These microorganisms work in a complex and coordinated manner to rapidly colonize and digest plant material^6^. Notable among the bacteria in the rumen are species such as *Fibrobacterium*^7^, *Ruminococcu* and *Butyrivibrio*^8,9^. Additionally, *Prevotella*, a genus involved in the degradation of non-cellulosic plant fibres, also populates the rumen in large numbers. Nonetheless, the precise mechanism that accounts for buffalo possessing a greater digestive capacity compared to cattle remains unclear. A recent study on macrogenomics also found that the microbiota in buffalo rumen possess a higher capability for fiber breakdown and produce less methane compared to that of dairy rumen microbiota^10^. Consequently, the structure and role of the rumen microbiota could differ between buffalo and cattle.

Those microbes work together synergistically and contribute substantially to the nutrient utilization, metabolism, growth performance, and health of the host^11^. Various elements, including nutrition, age, gender, breed, and geographic distribution, directly or indirectly influence rumen microorganisms^12,13^. Prior studies have predominantly concentrated on the dietary influences on microbial populations^2,14,15^, thereby leaving the impact of sex on rumen fermentation relatively unexplored. Studies have demonstrated that ruminants exhibit notable sex differences in the composition of their gut microbiota. These differences are evident not only in the types of microorganisms present but also in their abundance and functionality^16^. For instance, the abundance of specific bacterial populations varies significantly between male and female animals, potentially relating to sex-related physiological characteristics and metabolic processes^17^. The impact of sex on the microbiome may be influenced by a variety of factors, including hormone levels, genetic background, and dietary habits of the animal. Gender may affect the diversity and function of these microorganisms, thereby indirectly affecting animal health and performance^18,19^. The nutritional needs of an animal can differ across various physiological stages, potentially influencing the diversity and number of microorganisms present in the rumen. As a result, particular physiological and biological processes associated with reproduction and nurturing can greatly affect the microbial ecology of female buffaloes when compared to their male counterparts. This relationship highlights the necessity of considering sex-specific aspects in studies of the gastrointestinal microbiome in buffalo and similar species^2^. However, our understanding of the effect of sex on rumen microbes remains incomplete. Previous studies have primarily focused on the differences in feed digestibility between sexes^20,21^, but the sex-related variations in rumen microbial community structure and function have been largely overlooked. The emergence of high-throughput sequencing and bioinformatics has marked a new genomic era in rumen microbiome research, offering powerful tools for investigating these influences.

This study aims to investigate the effects of sex on rumen microorganisms, to dissect differences in microbial composition, diversity and functionality between sexes, and to elucidate the underlying physiological mechanisms. Furthermore, we aim to synthesize recent advancements in the understanding of sex-induced influences on rumen microorganisms and provide valuable insights for related areas of research.

## Materials and methods

### Experimental animals and sample

In a family farm in Dongzhi County, Anhui Province, ten healthy Dongliu buffaloes with an average body weight of 330.1 kg (± 8.98 kg) were selected. These buffaloes, matched for age and feeding mode, were categorized into four males and six females based on their sex. Under a ‘grazing supplementary’ feeding regimen, their diet primarily comprised maize stover, sweet elephant grass, and rice straw, complemented by a daily supplement of 1750 g of maize per buffalo. All buffaloes were fasted for 24 h and water-deprived for 8 h after the end of the test period following the conclusion of the test period. Subsequently, the buffaloes were slaughtered. Rumen fluid was collected and immediately frozen and stored at -80℃ until further processing. This fluid was preserved for DNA extraction and analysis of the bacterial community present in each animal.

### Total DNA extraction and metagenomic sequencing

Genomic DNA from Dongliu buffalo samples was extracted using the cetyltrimethylammonium bromide (CTAB) method, a standard technique in molecular biology, following the protocol provided by the manufacturer of the CTAB kit. Subsequently, the level of DNA degradation, potential contamination, and DNA concentration were assessed using the Agilent 5400, a reliable instrument for these measurements. Only the libraries that met our quality criteria were selected for pooling. Individual libraries were constructed using the NEBNext Ultra DNA Library Prep Kit and subsequently sequenced on the Illumina NovaSeq 6000 platform employing a 2 × 150 bp paired-end read protocol. The decision to use PE150 was based on both the effective library concentration and the quantity of data necessary for thorough analysis.

### Sequencing data analysis

In this project, we employed the Illumina NovaSeq platform to conduct paired-end sequencing of our samples. For further details on the sequencing format, please refer to the Fastq specification (https://en.wikipedia.org/wiki/Fastq). To ensure the accuracy and reliability of subsequent analysis, it is necessary to preprocess the raw sequencing data. This involves quality control using Trimmomatic with the following parameters^22^: ILLUMINACLIP: adapters path:2:30:10 SLIDINGWINDOW:4:20 MINLEN:50, and the removal of host sequences with Bowtie2^23^ using the—very sensitive setting. These steps are crucial to obtain valid sequences, or clean data, suitable for subsequent analysis.

### Bioinformatics analysis

MEGAHIT software^24^ was employed to assemble clean reads after the removal of host genes, resulting in the generation of contigs. Gene prediction within these contigs was performed using Prodigal software^25^, with the metagenomic pattern parameter set to '-p meta’. To establish a non-redundant set of genes, Cd-hit software^26^ was utilized, applying a global sequence identity threshold of '-G 1’ and a sequence identity cutoff of '-c 0.95’. The quantification of the non-redundant genes was carried out using Salmon software^27^, with parameters adjusted to enhance sensitivity and specificity: '–validate Mappings’ and '–meta’ for metagenomic mode. The translation of non-redundant genes into protein sequences was conducted using the Transeq command of Emboss software, preparing these sequences for subsequent BLAST and annotation processes^28^. The non-redundant protein sequences underwent BLAST searches against the EggNOG database^29^ to obtain KEGG, GO, and COG annotation information, utilizing Eggnog-mapper software with a stringent e-value cutoff of '–seed ortholog evalue 0.00001’. Additionally, these sequences were queried against the CAZyme database for functional annotation using DIAMOND software^28^, with parameters set to '-e 0.00001’ for the e-value threshold, '–id 80’ for the identity threshold, and '–top 3’ to ensure that only the top hits with bit scores at least 3% of the maximum score were included.

### Statistical analysis

All experiment data were collated and preliminary processed using Microsoft office Excel 2021.The differences in rumen microorganisms between male and female Dongliu buffaloes were statistically analyzed using SPSS software version 25.0. T-tests were conducted on data that exhibited a normal distribution, while non-parametric tests were applied to other datasets, with p < 0.05 as the significance threshold. Results for each group are presented as the "mean ± standard error" to ensure clarity and precision in the data representation.

## Results

### Sequencing data

Metagenomic direct sequencing downstream processing generated a total raw data volume of 64.57 GB, comprising 2,152,254,767 raw sequences (Table S1). Following quality control procedures and de-hosting, we retained 203,796,013 clean entries, averaging 20.38 million entries per sample. De novo assembly yielded an average of 315,154 contigs per sample, with an N50 value of 1,382 base pairs, indicating robust assembly quality. Quality assessments revealed that the valid sequences, defined by Q20 and Q30 metrics, exceeded 97.00% and 92.00% respectively. These high percentages reflect the high reliability and accuracy of the sequencing data, where Q20 and Q30 represent the proportions of sequence data with a base call accuracy of at least 99% and 99.9%, respectively.

In the present study, taxonomic classification was conducted using DIAMOND software (version 0.8.35) to map the sequences of a non-redundant gene catalog against the NR database, applying an e-value cutoff of 1 × 10^−5^. A total of 964 genera across 52 phyla were identified in the rumen samples. Bacteria dominated the at the kingdom level classification (97.53% of all collected samples), followed by archaea (1.87%), eukaryotes (0.16%) and viruses (0.43%) (Table 1, Table S2).

**Table 1:** Proportion of microorganisms at the level of the kindom.

Categories	n	Bacteria (%)	Archaea (%)	Fungi (%)	Viruses (%)
All	10	97.53 ± 0.37	1.87 ± 0.32	0.16 ± 0.03	0.43 ± 0.06
Female	6	97.88 ± 0.45	1.58 ± 0.36	0.15 ± 0.03	0.41 ± 0.10
Male	4	97.02 ± 0.61	2.32 ± 0.59	0.19 ± 0.05	0.47 ± 0.06

### Microbial diversity and taxonomic composition of rumen microorganisms in Dongliu Buffalo

**Fig. 1:**
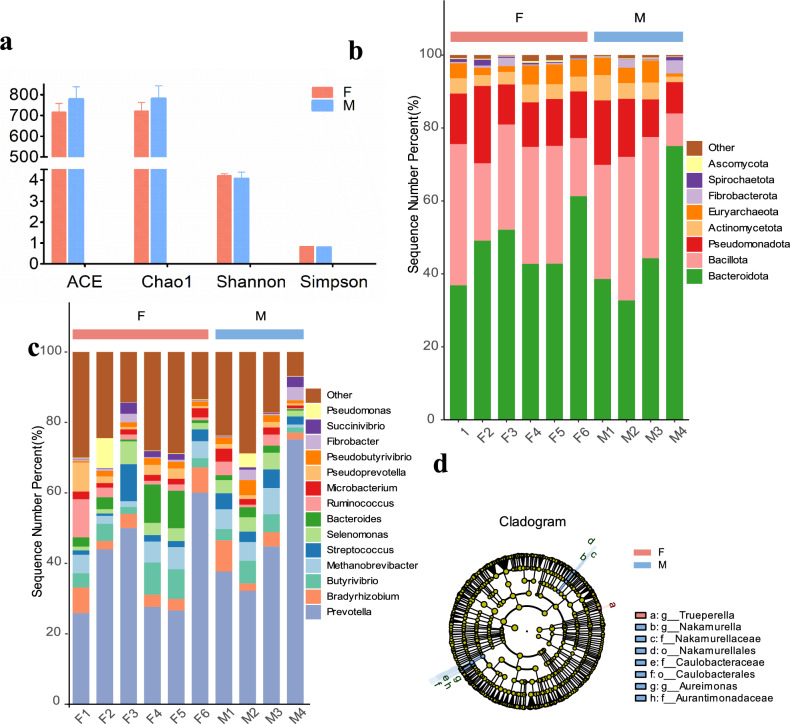
Diversity and compositional profiles of ruminal microbiota in female and male Dongliu buffalo. (**a**) Alpha diversity indices were compared between F and M groups using a T-test. Significance levels are denoted by *P < 0.05 and **P < 0.001. The abscissa labels group categories, and the ordinate represents mean diversity values. (**b**,**c**) Stacked bar plots display the relative abundance of rumen microbiota at the phylum (**b**) and genus (**c**) levels. (**d**) LEfSe (Linear Discriminant Analysis Effect Size) cladogram illustrating taxonomic differences between Females and Males. The six concentric circles correspond to phylum, class, order, family, genus and species. Colored nodes indicate taxa with significant enrichment (LDA score > 2, *P* < 0.05) in either group (Females: red; Males: blue), while light yellow nodes denote non-discriminatory taxa. Node size reflects the relative abundance of each taxon.

The alpha diversity of rumen microbiota, assessed via ACE, Chao1, Shannon, and Simpson indices, showed no significant differences between female and male buffaloes (T-text, all *P* > 0.05; Fig. 1a, Table S3). Beta diversity analysis based on Bray–Curtis distances and PCoA revealed broad similarity between groups (PERMANOVA, *P* = 0.82; Figure S1).

At the phylum level, *Bacteroidota* (47.54%) and *Bacillota* (28.20%), collectively accounting for 75.74% of the total sequences. Subdominant phyla included *Pseudomonadota* (13.63%), *Actinomycetota* (4.09%), and *Euryarchaeota* (3.89%), while low-abundance phyla (e.g., *Fibrobacterota, Spirochaetota*, and *Ascomycota*) each represented less than 1% of the community (Fig. 1b). Males exhibited marginally higher *Fibrobacterota* abundance (Females:0.68%, Males:1.61%, *P* = 0.05), while no significant sex differences were detected in dominant phyla (all *P* > 0.15) (Table S4).

At the genus level, the rumen microbiota was dominated by *Prevotella* (42.39%), followed by *Butyrivibrio* (4.73%) and *Bacteroides* (3.56%). Subdominant genera (e.g., *Bradyrhizobium*, *Methanobrevibacter*, *Streptococcus*) collectively accounted for less than 5% of the community (Fig. 1c). The abundance of *Bacteroides* was significantly higher in females (T-text, *P* = 0.01), whereas *Fibrobacter* exhibited a male-biased enrichment (T-text, *P* = 0.02). In contrast, no significant sex-associated variations were detected in dominant genera such as *Prevotella* (*P* = 0.76) or *Butyrivibrio* (*P* = 0.40) (Table S5). LDA identified significant taxonomic differences between sexes. Specific taxa were enriched in males (*Aurantimonadaceae, Aureimonas, Caulobacteraceae*) and females (*Trueperella*) (LDA > 2, *P* < 0.05; Fig. 1d).

## Functional analysis of rumen microorganisms in Dongliu Buffalo

### KEGG functional annotation

**Fig. 2:**
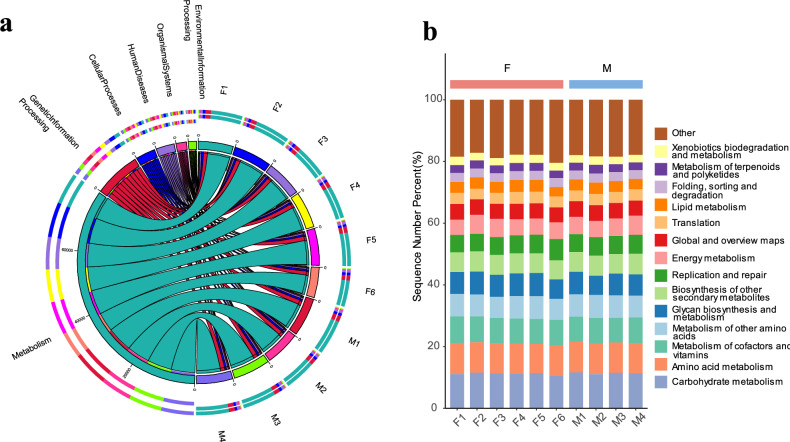
(**a**) Illustrates the relative abundance of KEGG in Dongliu Buffalo metagenome. The left half of the circle represents the functional abundance composition, while the right half corresponds to the sample. (**b**) Presents a differential analysis of KEGG pathways between male (M) and female (F) Dongliu buffaloes. Figures a and b are cited from the source: www.kegg.jp/kegg/kegg1.html.

A total of 961 non-redundant genes were mapped to 429 KEGG pathways (Table S6). Among the first functional hierarchy, the dominant pathways, defined as those with an average relative abundance exceeding 5% for at least one group, included “Metabolism” (70.88 ± 0.37%), "Genetic Information Processing" (12.91 ± 0.13%), “Cellular Processes” (5.46 ± 0.17%), and “Human Diseases” (5.07 ± 0.05%) (Fig. 2a). In the secondary pathways, the most abundant included “Carbohydrate Metabolism” (11.17 ± 0.09%), "Amino Acid Metabolism" (9.74 ± 0.06%), "Metabolism of Cofactors and Vitamins" (8.23 ± 0.07%), "Metabolism of Other Amino Acids" (7.15 ± 0.07%), "Glycan Biosynthesis and Metabolism" (6.97 ± 0.13%), and "Biosynthesis of Other Secondary Metabolites" (6.36 ± 0.05%), all of which had an average relative abundance greater than 6% for at least one group (Fig. 2b). In the carbohydrate metabolic pathway, specifically within Glycolysis/Gluconeogenesis (*ko00010*), the Pentose Phosphate Pathway (*ko00030*), Starch and Sucrose Metabolism (*ko00500*), Fructose and Mannose Metabolism (*ko00051*), Galactose Metabolism (*ko00052*), Amino Sugar and Nucleotide Sugar Metabolism (*ko00520*), and Pyruvate Metabolism (*ko00620*), we have identified the metabolic routes involved in cellulose degradation. Enzymes such as EC3.2.1.4 (endoglucanase), EC3.2.1.1 (beta-1,4-exoglucanase), and EC3.2.1.20 (beta-1,4-endoglucanase) directly participate in the breakdown of cellulose by cleaving glycosidic bonds within the cellulose polymer chains. Additionally, EC3.2.1.52 (xylanase), EC3.2.1.23 (alpha-L-arabinofuranosidase), and EC3.2.1.21 (beta-1,4-galactosidase) regulate the degradation of hemicellulose, another component of the plant cell wall that is often associated with cellulose. These enzymes are integral to the metabolic pathways related to carbohydrate metabolism and play significant roles in the efficient breakdown of cellulosic biomass. LEfSe analysis identified three EC numbers (EC2.4.1.20, EC1.2.1.90, and EC2.7.1.58) as significantly enriched biomarkers in male buffalo. (LDA > 2, *P* < 0.05; Figure S2).

### Carbohydrate-active enzymes functional annotation

**Fig. 3:**
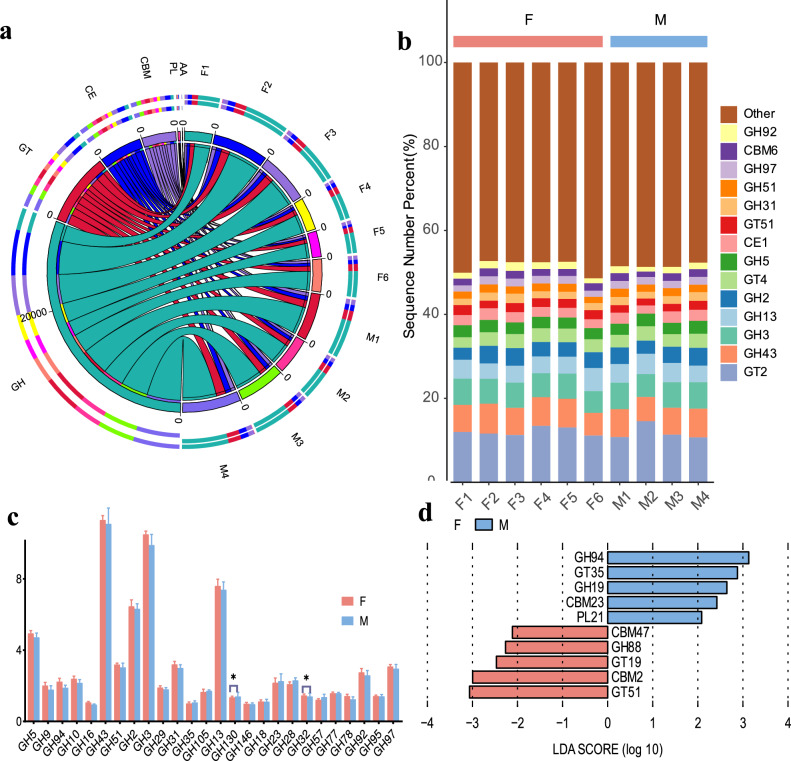
(**a**) Illustrates the relative abundance of CAZymes in Dongliu Buffalo metagenome. The left half of the circle represents the functional abundance composition, while the right half corresponds to the sample. (**b**) Abundance of carbohydrate-active enzyme genes in the rumen metagenome of Dongliu buffaloes. The Y–axis is for different GH families, X–axis is the abundance of GH. (**c**) The composition of GH families with relative abundances exceeding 0.01%. (**d**) Utilize Linear discriminant analysis (LDA) effect size analysis (LEFSe) to identify CAZyme with significant differences in relative abundance between sex groups. Taxa enriched in the male group are indicated with a positive LDA score (blue), and taxa enriched in the female group have a negative score (red). Only taxa with an LDA score > 2 and a P value < 0.05 are shown.

In the present study, we identified a total of 462 genes encoding class-level carbohydrate-active enzymes. As illustrated in Fig. 3a, Glycoside hydrolases (GHs, 62.84%, 63.38%) were the most abundant, followed by glycosyltransferases (GTs, 17.5%, 17.32%), carbohydrate esterases (CEs, 10.29%, 10.45%) and carbohydrate-binding modules (CBMs, 8.71%, 8.26%). A smaller proportion of genes was associated with polysaccharide lyases (PLs, 0.59%, 0.52%) and auxiliary activities (AAs, 0.59%, 0.52%) (Table S7). However, there was no significant difference in the abundance of GH CAZymes between the two hosts (*P* = 0.908). CAZymes belonging to the GT class were the second most abundant in the buffalo rumen metagenome, yet the distribution frequency between the two hosts was comparable (*P* = 0.999). Similarly, the abundances of other CAZymes, such as CE, CBM, and PL, were also comparable between males and females.

At the family level, the top 15 most abundant are GT2, GH43, GH3, GH2, GT4, GH13 GH5, CE1, GT51, GH31, GH51, GH97, CBM6, and GH92(Fig. 3b). The enzymes from families GH5, GH3, GH43, GH13, GT4, and GT2 are particularly important for the degradation of hemicellulose, while GH97 may have a role in cellulose degradation under specific conditions (Fig. 3b). CAZymes often exhibit a linked modular structure known as CBMs, which helps in adhesion to carbohydrates. In total, 11 CBMs were annotated in the rumen microbiome that associate with both cellulase and hemicellulose. Additionally, 4 and 15 CBMs exclusively associating with cellulase and hemicellulose genes, respectively, were also found in the rumen microbiome. CBM2, CBM6, CBM13, CBM32, and CBM36 were found to be the most abundant, covering 23–28% of the CBMs (Table S8). In Fig. 3c, we analyzed the composition of GH families with relative abundances exceeding 0.01%. The ranking of these GH families based on their abundance is as follows: GH43, GH3, GH13, GH2, GH5, GH31, GH51, GH97, GH92, GH10, GH28, GH23, GH94, GH9, GH29, GH105, GH77, GH32, GH95, GH130, GH78, GH57, GH18, GH35, GH16, and GH146. LEfSe analysis revealed that among the genes encoding CAZymes involved in the deconstruction of cellulose, hemicellulose, starch, protein, and lignin, the following were enriched: CBM47, GH88, GT19, CBM2, and GT51 in the female group, while GH94, GT35, GH19, CBM23, and PL21 were enriched in the male group (LDA > 2, *P* < 0.05; Fig. 3d). GH94, GH19, CBM23, CBM47, CBM2, GT19, and GT51 are implicated in cellulose degradation. In contrast, GH88 is associated with hemicellulose degradation, while GH94 also participates in the degradation of oligosaccharides.

As shown in Table 2, a significant amount of fiber-degrading enzymes capable of degrading cellulose and hemicellulose is present in the rumen of Dongliu buffalo. The Dongliu buffalo exhibits a notably high proportion of cellulase gene families GH5 and GH9, which are core enzymes for cellulose degradation. The GH10 family (endoxylanase) shows a similar proportion across different breeds (2.01–2.64), indicating strong evolutionary conservation of this gene family in ruminants. GH43 and GH51 are key enzymes for the degradation of xylan and arabinoxylan, and their uniqueness may reflect the co-evolutionary characteristics of the gut microbiome and the host in Dongliu buffalo. In the oligosaccharide degradation-related families, the Dongliu buffalo exhibited a higher proportion of GH28 (2.18 vs. 0.21–1.93) and GH35 (1.04 vs. 0.49–0.78). However, the proportion of GH2 in the GH families of Dongliu buffalo is lower than that found in other cattle breeds. Conversely, the proportion of GH3 is lower than that in Indian buffalo but higher than that in Haizi buffalo and Mongolian cattle.

**Table 2:** Distribution of GH genes in the rumen microbial metagenomes of different cattle breeds.

	Gene family	Dongliu buffalo	Indian buffalo^30^	Haizi buffalo^31^	Mongolian cattle^32^
Cellulase	GH5	4.86	1.50	2.88	0.20
	GH9	1.92	0.26	1.89	1.30
Hemicellulase	GH10	2.31	2.64	2.01	2.20
	GH43	11.22	–	–	–
	GH51	3.13	–	–	–
Oligosaccharide-degrading	GH2	6.41	9.85	9.69	11.20
	GH3	10.26	18.30	4.81	5.03
	GH28	2.18	0.21	1.69	1.93
	GH29	1.87	2.28	2.06	1.70
	GH35	1.04	0.78	0.49	0.67
	GH78	1.36	2.85	–	–

### COG functional annotation

**Fig. 4:**
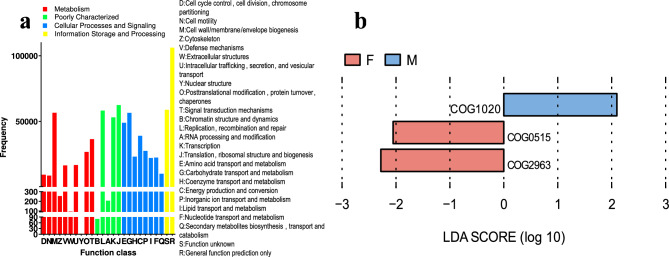
(**a**) Presents the COG functional annotation distribution diagram for genes identified in the rumen microbiota of Dongliu buffalo. (**b**) Utilize Linear discriminant analysis (LDA) effect size analysis (LEFSe) to identify COG with significant differences in relative abundance between sex groups. Taxa enriched in the male group are indicated with a positive LDA score (blue), and taxa enriched in the female group have a negative score (red). Only taxa with an LDA score > 2 and a P value < 0.05 are shown.

**Fig. 5:**
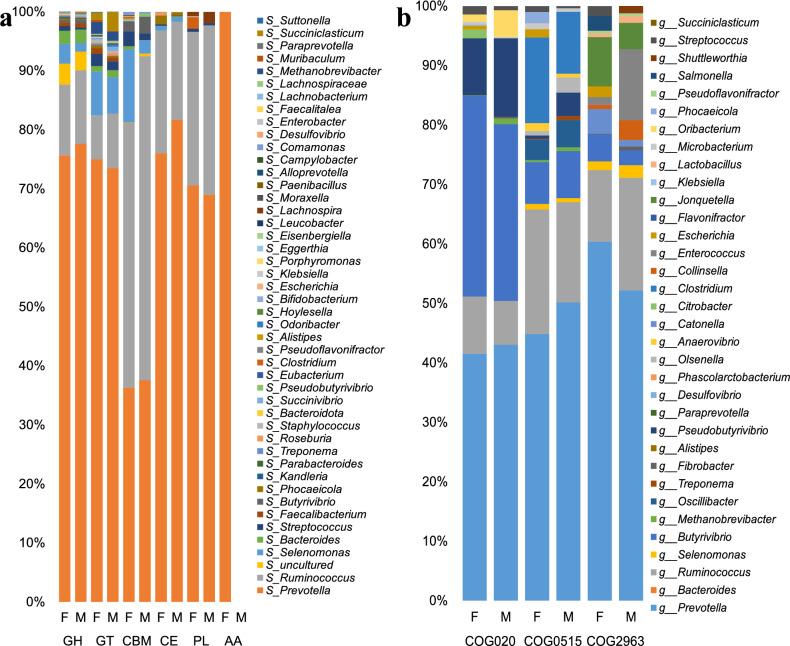
(**a**,**b**) Relative contribution of different taxa (species, genus levels) to identified rumen-enriched functional attributes of CAZymeme and COG encoded genes.

A cluster of orthologous groups of proteins (COG) annotation was conducted for 6,268,593 non-redundant genes using Diamond (version 0.8.35), based on the eggNOG database with an e-value cutoff of 1 × 10 − 5. This analysis yielded a total of 4,387 COG functional genes. The majority of these genes were categorized under General function prediction only (R), Translation, ribosomal structure and biogenesis (J), and Replication, recombination and repair (L) in the COG database (Fig. 4a). The number of genes involved in carbohydrate transport and metabolism (G), totaling 56,524.39, is significantly higher than that of certain core metabolic categories, such as lipid metabolism (I: 22,170.52). However, it is lower than that of energy metabolism (C: 38,993.48) and secondary metabolism (Q: 10,157.15). This observation underscores the crucial role of carbohydrate metabolism in central metabolic processes. LEfSe analysis revealed that information storage and processing (COG0515) and Replication, recombination and repair (COG2963) were more prevalent in female buffalo compared to male buffalo, whereas Secondary metabolites biosynthesis, transport and catabolism (COG1020) was found to be more abundant in group M (LDA > 2, *P* < 0.05; Fig. 5b).

### Functional contribution analysis

At the species level, we identified significant linkages between rumen microorganisms and CAZymeme families in Dongliu buffalo (Fig. 5a). *Prevotella* dominated across five CAZymeme classes (GH, GT, PL, CE, AA), with GH-family enzymes—primarily sourced from *Prevotella, Ruminococcus, Selenomonas, Bacteroides,* and *Streptococcus*—representing the core cellulolytic machinery. Carbohydrate-binding modules (CBMs), critical for substrate adhesion, were predominantly associated with *Ruminococcus, Prevotella,* and *Selenomonas*. The genus-level analysis revealed the distinct contributions of microorganisms to the functional profiles of Clusters of Orthologous Groups (COG), with the genus *Prevotella* identified as the primary contributor to these functions (Fig. 5b). The cellulolytic bacterial genera *Prevotella*, *Butyrivibrio*, and *Ruminococcus* were identified as primary contributors to COG020, COG0515, and COG2963 functional categories, respectively. Comparative analysis between groups demonstrated differential microbial associations: in COG020, *Selenomonas* exhibited marginally higher abundance in female buffalo compared to group M (*P* = 0.07). COG0515 showed significant enrichment of *Ruminococcus* in female buffalo relative to male buffalo (*P* < 0.01), suggesting enhanced fibrolytic capacity in the former. For COG2963, *Jonquetella* displayed a non-significant trend toward increased abundance in female buffalo (*P* = 0.12). These findings highlight sex-specific functional partitioning within the rumen microbiome, particularly in lignocellulose degradation pathways.

## Discussion

The objective of this study was to investigate the functional and taxonomic characteristics of rumen microbial populations, as well as the impact of sex on these microorganisms in Dongliu buffalo, utilising macrogenomic techniques. The rumen of ruminants hosts a diverse array of microorganisms, including bacteria, archaea, and fungi^33^. Throughout the development of the animals, rumen microorganisms establish a symbiotic relationship with their hosts, playing a crucial role in the structure, function, and immunity of the rumen. Our metagenomic analysis of rumen microorganisms in Dongliu buffalo showed that bacteria were the most abundant microbiota (97%), followed by archaea. This is consistent with previous findings that more than half of the rumen microorganisms are bacteria, which play an important role in host nutrition, physiology and immunity^34^. The abundance of bacteria in the rumen indicates that the rumen ecosystem is capable of fermenting and digesting complex polysaccharides, including cellulose, hemicellulose and lignin. This process is essential for the efficient extraction of energy from feed for host animals^6^. It is currently established that Firmicutes and Bacteroidetes are the predominant phyla in the gastrointestinal microbiota of ruminants^35–38^. The results of this study indicate that the abundance of *Bacteroidota* and *Bacillota* in Dongliu Buffalo accounts for 47.54% and 28.20% of the total microbial biomass, respectively, making them the most dominant phyla. This finding aligns with previous studies that have reported the dominance of these phyla in the rumen flora of ruminants^33^, where these phyla are central to lignocellulose degradation and short-chain fatty acid production^39^. This phenomenon may be linked to the forage consumed, as crop straw primarily consists of fiber. Previous research has shown that *Bacillota* in the gastrointestinal tract can effectively decompose cellulose and lignin, while *Bacteroidota* serve as the main degraders of non-fibrous plant components in the rumen^16^. The observed variations may be influenced by several factors, including breed, age, and feeding methods of the animals. The alpha diversity indices (ACE, Chao1, Shannon, and Simpson) showed no significant differences between female and male buffaloes, indicating similar microbial diversity in both sexes. This finding aligns with studies on Tibetan goats, which reported no significant sex differences in rumen microbial diversity^13^. However, it contrasts with research on blue sheep, where sexual dimorphism was observed in microbiota composition^16^. Factors such as age, breed, gender, and geographical environment significantly influence the composition of the rumen flora^40,41^. Sex has some influence on the composition and number of microorganisms in the rumen of buffalo. A comparison of the rumen flora between female and male Dongliu buffaloes revealed no significant differences in the overall composition of the rumen flora based on gender. However, notable differences were observed in the structural composition of the rumen flora. Specifically, the abundance ratios of Firmicutes and Bacteroidetes did not differ significantly between the sexes. At the phylum level, *Bacteroidota* and *Bacillota* were the dominant groups. This is similar to findings in other ruminants, such as cattle and Holstein cows, where Bacteroidota and Bacillota are also dominant^36,42^. The high abundance of *Bacteroidota* and *Bacillota* is likely due to their ability to degrade complex plant polymers, which is essential for the digestion of fibrous feed in ruminants^36,42^. At the genus level, *Prevotella* (42.39%) was the most abundant, followed by Butyrivibrio (4.73%) and Bacteroides (3.56%). The dominance of *Prevotella* is a common feature in ruminant microbiomes, as it plays a key role in cellulose and hemicellulose degradation^7,8^. However, the higher abundance of *Bacteroides* in females (*P* = 0.01) and Fibrobacter in males (*P* = 0.02) highlights sex-specific differences in microbial composition. such as hormonal fluctuations or dietary intake patterns^43^. Our findings align with broader ruminant microbiota studies, where *Bacteroidota* and *Bacillota* dominate the rumen across cattle, sheep, and goats^44^. *Prevotella* and *Ruminococcus*—core cellulose degraders—exhibited male-biased dominance in GH and CBM activities (*P* < 0.05), suggesting enhanced cellulose binding and hydrolysis efficiency in male microbiomes. The sex-associated enrichment of *Fibrobacterota* in males parallels observations in goats, where males harbor higher fibrolytic taxa under high-fiber diets^45^. Conversely, the absence of significant differences in dominant genera (*Prevotella, Butyrivibrio*) between sexes diverges from studies in dairy cows, where lactation stage—a sex-linked physiological state—drives pronounced microbial shifts^46^. These discrepancies may arise from methodological differences (e.g., sequencing depth) or host-specific ecological dynamics. Conversely, the female-biased *Bacteroides* could relate to immune-modulatory roles, as estrogen has been shown to enhance mucosal glycan utilization by *Bacteroides spp*. in mammals^47^. Additionally, the male-enriched taxa (*Aurantimonadaceae, Aureimonas*) and female-associated *Trueperella* (LDA > 2) may reflect sex-driven immune or hormonal interactions, as *Trueperella* is linked to mucosal colonization in estrogen-rich environments^48^.

The connection between microorganisms and host cells is primarily reflected in the provision of direct substrates within the microbial food chain, with rumen microorganisms playing a crucial role in the absorption and metabolism of nutrients^49^. In the KEGG analysis, we found that the rumen microorganisms of Dongliu buffalo play a significant role in the digestive and metabolic processes of the host animal, particularly in carbohydrate breakdown and energy metabolism. The functional analysis revealed that “Metabolism” (70.88 ± 0.37%) was the dominant pathway, with carbohydrate metabolism being the most abundant secondary pathway. This is consistent with the crucial role of carbohydrates in ruminant digestion^6^. The identification of enzymes such as EC3.2.1.4 (endoglucanase) and EC3.2.1.52 (xylanase) involved in cellulose and hemicellulose degradation underscores the importance of these enzymes in fiber breakdown. Key pathways such as Glycolysis/Gluconeogenesis (*ko00010*) and Starch and Sucrose Metabolism (*ko00500*) were enriched, supported by high abundances of cellulolytic enzymes (e.g., *GH5, GH9, GH43*) and hemicellulases (e.g., *GH10, GH51*). The identification of 462 CAZymes underscores the microbiome’s adaptability to fibrous diets, with glycoside hydrolases (GHs, ~ 63%) dominating, consistent with their role in hydrolyzing glycosidic bonds in cellulose and hemicellulose^50^. Sex-specific differences were subtle but notable: females exhibited enrichment in *CBM47*, *GT19*, and *GT51* (linked to cellulose adhesion and modification), whereas males showed higher *GH94, PL21*, and *CBM23* (associated with oligosaccharide and lignin-related degradation), potentially tied to divergent energy demands or hormonal regulation^51^. COG analysis further highlighted the centrality of carbohydrate metabolism (56,524 genes), surpassing lipid and secondary metabolism, emphasizing its role in sustaining rumen fermentation. The female-biased prevalence of Replication, recombination, and repair (COG2963) genes may reflect microbial community stability under hormonal fluctuations, while male-enriched secondary metabolite biosynthesis (COG1020) could relate to niche competition or detoxification^52^**.** The dominance of GH families (*GH5, GH9, GH43*) aligns with findings in cattle and goats, where these enzymes drive fiber degradation^53^. However, Dongliu buffalo’s higher *GH28* abundance compared to Indian buffalo and cattle (2.18% vs ≤ 1.93%) suggests host-specific adaptations to regional diets rich in pectin or oligosaccharides^33^**.** Sex-linked CAZymeme differences mirror observations in dairy cows, where lactation-driven metabolic demands alter microbial enzyme profiles^47^. This discrepancy may stem from Dongliu buffalo’s uniform feeding practices or genetic buffering against sex-driven microbial shifts. The microbiome’s emphasis on carbohydrate metabolism likely reflects co-evolution with the host’s high-fiber diet, optimizing energy harvest from recalcitrant plant biomass^6^**.** The prominence of *GH5* and *GH9* aligns with their dual activity on crystalline cellulose, a trait critical for survival in low-digestibility environments^54^. The unique *GH28* enrichment in Dongliu buffalo may stem from selective pressure to degrade pectin-rich forages common in tropical regions, a hypothesis supported by similar adaptations in water buffalo^11^. Sex-specific CAZymeme patterns (e.g., female-enriched *GT51*) may arise from estragon’s modulation of mucosal glycosylation, favoring taxa with adhesive CBMs (*CBM47*) to colonize estrogen-primed epithelia^55^. Conversely, male-enriched *GH94* (cellobiose phosphorylase) could enhance energy yield from cellulose under testosterone-driven anabolic demands^56^. This study delineates the functional landscape of the Dongliu buffalo rumen microbiome, highlighting its exceptional capacity for lignocellulose degradation and subtle sex-linked enzymatic specialization. Carbohydrate metabolism dominates, driven by *GH5, GH9*, and *GH43*, enabling efficient fiber digestion. Host-specific adaptations (e.g., *GH28* abundance) reflect dietary and evolutionary pressures. Sex-specific CAZymes suggest hormonal or metabolic modulation of microbial functions. The doubled GH and CBM abundances in male *Butyrivibrio* further indicate synergistic secretion of fibrolytic enzymes for crystalline cellulose decomposition. In contrast, female-enriched *Selenomonas* dominated GH and CE activities, likely specializing in hemicellulose side-chain modification (e.g., acetyl ester hydrolysis). Male *Succiniclasticum* showed elevated GT and CE activities, implicating roles in oligosaccharide recycling from fiber-derived substrates. At the genus level, *Prevotella, Butyrivibrio*, and *Ruminococcus* were primary contributors to COG functional categories linked to carbohydrate metabolism (COG020), replication/repair (COG0515), and recombination (COG2963). Female buffalo exhibited significant enrichment of *Ruminococcus* in COG0515 (*P* < 0.01), associated with enhanced fibrolytic capacity, while *Jonquetella* showed a non-significant trend toward female bias in COG2963. These findings underscore sex-driven microbial niche specialization: males prioritize GH-CBM systems for high-fiber digestion, whereas females rely on auxiliary enzymes (CE/PL) to maintain metabolic flexibility. The dominance of *Prevotella* and *Ruminococcus* in cellulose degradation aligns with their established roles in ruminants, where they drive lignocellulose breakdown via *GH48* and *GH9* enzymes^7^. However, the male-specific enrichment of *Prevotella*-GH/CBM contrasts with dairy cow studies, where lactation stage—not sex—drives microbial shifts^15^. Similarly, the female-biased *Selenomonas*-CE activity mirrors findings in goats, where estrogen upregulates esterase-linked hemicellulose modification^57^.

## Conclusions

In conclusion, the present study provides a comprehensive analysis of the rumen microbiota in Dongliu buffalo, highlighting the dominant bacterial groups and their functional roles in fiber degradation. The findings suggest that sex-specific differences in microbial composition and function may influence rumen fermentation and nutrient utilization. However, the study has some limitations. The sample size was relatively small, and the study was conducted under specific feeding conditions, which may limit the generalizability of the results. Future research should focus on larger sample sizes and explore the impact of different diets and environmental factors on rumen microbiota. Additionally, meta transcriptomic and metabolomic analyses could provide deeper insights into the functional activities of the rumen microbiome and their relationship with host physiology.

## Supplementary Information

Supplementary Information 1.

Supplementary Information 2.

Supplementary Information 3.

Supplementary Information 4.

Supplementary Information 5.

Supplementary Information 6.

Supplementary Information 7.

Supplementary Information 8.

Supplementary Information 9.

## Data Availability

The genome assemblies generated in this study are available in the NCBI under BioProject IDs of PRJNA1131156. https://dataview.ncbi.nlm.nih.gov/object/PRJNA1131156?reviewer=t1vjs45ged4gtv8vmve0d787fr.

## References

[1] Jia, Y., Zhao, S., Xu, L., Cheng, Z. & He, Z. Countermeasures for the conservation, development, and utilisation of Dongliu buffalo breed resources. *China Herbivore Sci.* **34**, 66 (2014).

[2] Pu, X. X. et al. Comparison of in situ ruminal straw fiber degradation and bacterial community between buffalo and Holstein fed with high-roughage diet. *Front. Microbiol.* **13**, 1079056 (2022). 36699590 10.3389/fmicb.2022.1079056 PMC9868309

[3] Malik, P. K. et al. Comparative rumen metagenome and CAZymeme profiles in cattle and buffaloes: implications for methane yield and rumen fermentation on a common diet. *Microorganisms* **1**, 1 (2024). 10.3390/microorganisms12010047 PMC10818812 38257874

[4] Di Stasio, L. & Brugiapaglia, A. Current knowledge on river buffalo meat: a critical analysis. *Animals-Basel* **11**, 1 (2021). 10.3390/ani11072111 PMC8300413 34359238

[5] Vargas-Ramella, M. et al. Buffalo milk as a source of probiotic functional products. *Microorganisms* **9**, 1 (2021). 10.3390/microorganisms9112303 PMC8620832 34835429

[6] Mizrahi, I., Wallace, R. J. & Morais, S. The rumen microbiome: balancing food security and environmental impacts. *Nat. Rev. Microbiol.* **19**, 553 (2021). 33981031 10.1038/s41579-021-00543-6

[7] Palevich, N. et al. Comparative genomics of Rumen butyrivibrio spp. uncovers a continuum of polysaccharide-degrading capabilities. *Appl. Environ. Microb.* **86**, e01919 (2019). 10.1128/AEM.01993-19 PMC6912079 31653790

[8] Liang, J. et al. Metagenomic analysis reveals the efficient digestion mechanism of corn stover in Angus bull rumen: Microbial community succession, CAZymeme composition and functional gene expression. *Chemosphere* **336**, 139242 (2023). 37330070 10.1016/j.chemosphere.2023.139242

[9] Sengupta, K. et al. Genomic architecture of three newly isolated unclassified Butyrivibrio species elucidate their potential role in the rumen ecosystem. *Genomics* **114**, 110281 (2022). 35124176 10.1016/j.ygeno.2022.110281

[10] Tong, F. et al. The microbiome of the buffalo digestive tract. *Nat. Commun.* **13**, 823 (2022). 35145088 10.1038/s41467-022-28402-9 PMC8831627

[11] Wu, X. et al. Characterizing the alteration in rumen microbiome and carbohydrate-active enzymes profile with forage of muskoxen rumen through comparative metatranscriptomics. *Microorganisms* **10** (2022). 10.3390/microorganisms10010071 PMC8777777 35056520

[12] Furman, O. et al. Stochasticity constrained by deterministic effects of diet and age drive rumen microbiome assembly dynamics. *Nat. Commun.* **11**, 1904 (2020). 32312972 10.1038/s41467-020-15652-8 PMC7170844

[13] Guo, X. et al. Sex differences in rumen fermentation and microbiota of Tibetan goat. *Microb. Cell Fact* **21**, 55 (2022). 35392919 10.1186/s12934-022-01783-8 PMC8991483

[14] Ampapon, T., Wanapat, M. & Kang, S. Rumen metabolism of swamp buffaloes fed rice straw supplemented with cassava hay and urea. *Trop. Anim. Health Pro.* **48**, 779 (2016). 10.1007/s11250-016-1026-5 26898691

[15] Iqbal, M. W. et al. Comparative study of rumen fermentation and microbial community differences between water buffalo and Jersey cows under similar feeding conditions. *J. Appl. Anim. Res.* **46**, 740 (2018).

[16] Zhu, Z. et al. Seasonal variation and sexual dimorphism of the microbiota in wild blue sheep (Pseudois nayaur). *Front. Microbiol.* **11**, 1260 (2020). 32670222 10.3389/fmicb.2020.01260 PMC7332577

[17] Guo, W. et al. Survey of the fecal microbiota of indigenous small ruminants living in different areas of Guizhou. *Front. Microbiol.* **15**, 1415230 (2024). 39176283 10.3389/fmicb.2024.1415230 PMC11340823

[18] Cholewinska, P., Szeligowska, N., Smolinski, J. & Bawej, M. Selected factors affecting the ruminant gastrointestinal microbiome and its basal composition. *Postepy Biochem.* **67**, 72 (2021). 34378897 10.18388/pb.2021_374

[19] Keum, G. B. et al. Understanding the diversity and roles of the ruminal microbiome. *J. Microbiol.* **62**, 217 (2024). 38662310 10.1007/s12275-024-00121-4

[20] He, Y. et al. Effects of the gender differences in cattle rumen fermentation on anaerobic fermentation of wheat straw. *J. Clean Prod.* **205**, 845 (2018).

[21] Tracy, M. & Howes-Mischel, R. Gender, microbial relations, and the fermentation of food. *Cuizine* **9**, 1 (2018).

[22] Bolger, A. M., Lohse, M. & Usadel, B. Trimmomatic: A flexible trimmer for Illumina sequence data. *Bioinformatics* **30**, 2114 (2014). 24695404 10.1093/bioinformatics/btu170 PMC4103590

[23] Langmead, B. & Salzberg, S. L. Fast gapped-read alignment with Bowtie 2. *Nat. Methods* **9**, 357 (2012). 22388286 10.1038/nmeth.1923 PMC3322381

[24] Li, D., Liu, C. M., Luo, R., Sadakane, K. & Lam, T. W. MEGAHIT: an ultra-fast single-node solution for large and complex metagenomics assembly via succinct de Bruijn graph. *Bioinformatics* **31**, 1674 (2015). 25609793 10.1093/bioinformatics/btv033

[25] Hyatt, D. et al. Prodigal: prokaryotic gene recognition and translation initiation site identification. *BMC Bioinformatics* **11**, 119 (2010). 20211023 10.1186/1471-2105-11-119 PMC2848648

[26] Li, W. & Godzik, A. Cd-hit: a fast program for clustering and comparing large sets of protein or nucleotide sequences. *Bioinformatics* **22**, 1658 (2006). 16731699 10.1093/bioinformatics/btl158

[27] Patro, R., Duggal, G., Love, M. I., Irizarry, R. A. & Kingsford, C. Salmon provides fast and bias-aware quantification of transcript expression. *Nat. Methods* **14**, 417 (2017). 28263959 10.1038/nmeth.4197 PMC5600148

[28] Buchfink, B., Xie, C. & Huson, D. H. Fast and sensitive protein alignment using DIAMOND. *Nat. Methods* **12**, 59 (2015). 25402007 10.1038/nmeth.3176

[29] Huerta-Cepas, J. et al. EGGNOG 5.0: A hierarchical, functionally and phylogenetically annotated orthology resource based on 5090 organisms and 2502 viruses. *Nucleic Acids Res.* **47**, D309 (2019). 10.1093/nar/gky1085 PMC6324079 30418610

[30] Singh, K. M. et al. High potential source for biomass degradation enzyme discovery and environmental aspects revealed through metagenomics of Indian Buffalo Rumen. *Biomed. Res. Int.* **2014**, 267189 (2014). 25136572 10.1155/2014/267189 PMC4124647

[31] Huimin, Z. et al. Metagenomic analysis of microorganisms in rumen of Haizi Buffalo. *Chin. J. Anim. Nutr.* **29**, 4151 (2017).

[32] Jianfei, M. et al. Macrogenomic analysis of rumen microorganisms in Mongolian cattle. *Chin. J. Anim. Sci. 1* (2025).

[33] Sanjorjo, R. A., Tseten, T., Kang, M., Kwon, M. & Kim, S. In pursuit of understanding the rumen microbiome. *Fermentation-Basel* **9** (2023).

[34] Flachowsky, G., Rumen Microbiology: Burk A Dehority (Ed.), Nottingham University Press, Nottingham, NG11 OAX, UK, 2003, Hardcover, ISBN 1-897676-99-9, £ 40, 372 pp. *Anim. Feed Sci. Tech.* **113**, 253 (2004).

[35] Ji, S. et al. Comparison of rumen bacteria distribution in original rumen digesta, rumen liquid and solid fractions in lactating Holstein cows. *J. Anim. Sci. Biotechnol.* **8**, 16 (2017). 28168037 10.1186/s40104-017-0142-z PMC5286850

[36] Ji, H. et al. A comparison of growth performance, blood parameters, rumen fermentation, and bacterial community of tibetan sheep when fattened by pasture grazing versus stall feeding. *Microorganisms* **12** (2024). 10.3390/microorganisms12101967 PMC11509657 39458276

[37] Guo, T. et al. Effects of the alpine meadow in different phenological periods on rumen fermentation and gastrointestinal tract bacteria community in grazing yak on the Qinghai-Tibetan Plateau. *BMC Microbiol.* **24**, 62 (2024). 38373936 10.1186/s12866-024-03182-y PMC10875897

[38] Zhao, Y., Guo, Y., Yang, C., Song, Z., & Luo, X. Differences in milk fatty acids profile of two breeds of water buffaloes explained by their gastrointestinal microbiota. *Animals-Basel* **14** (2024). 10.3390/ani14152146 PMC11311110 39123672

[39] Auer, E. et al. Horizontal metaproteomics and CAZymes analysis oflignocellulolytic microbial consortia selectively enriched from cow rumen and termitegut. *ISME Commun.* **3**, 134 (2024).

[40] Zhao, Y. et al. Effect of different genetic backgrounds on rumen microbiota and serum metabolic phenotypes in beef cattle. *Sci. Rep.-UK* **14**, 24005 (2024). 10.1038/s41598-024-74988-z PMC11473713 39402126

[41] Li, F. et al. Host genetics influence the rumen microbiota and heritable rumen microbial features associate with feed efficiency in cattle. *Microbiome* **7**, 92 (2019). 31196178 10.1186/s40168-019-0699-1 PMC6567441

[42] Malik, P. K. et al. Comparative rumen metagenome and CAZymeme profiles in cattle and buffaloes: implications for methane yield and rumen fermentation on a common diet. *Microorganisms* **12** (2023). 10.3390/microorganisms12010047 PMC10818812 38257874

[43] Bolnick, D. I. et al. Individual diet has sex-dependent effects on vertebrate gut microbiota. *Nat. Commun.* **5**, 4500 (2014). 25072318 10.1038/ncomms5500 PMC4279269

[44] Min, B. R. et al. Characterization of the ruminal microbiota in sheep and goats fed different levels of tannin-rich Sericea lespedeza hay. *J. Anim. Sci.* **102**, 198 (2024). 10.1093/jas/skae198 PMC11484804 39018107

[45] Guo, W. et al. Survey of the fecal microbiota of indigenous small ruminants living in different areas of Guizhou. *Front. Microbiol.* **15**, 1 (2024). 10.3389/fmicb.2024.1415230 PMC11340823 39176283

[46] Zhang, C. et al. An integrated microbiome- and metabolome-genome-wide association study reveals the role of heritable ruminal microbial carbohydrate metabolism in lactation performance in Holstein dairy cows. *Microbiome* **12**, 232 (2024). 39529146 10.1186/s40168-024-01937-3 PMC11555892

[47] Wang, Z. Y. & Gong, J. F. Gut microbiota and immune-related diseases. *Chin. J. Gastrointes. Surg.* **25**, 777 (2022). 10.3760/cma.j.cn441530-20211130-00480 36117368

[48] Qu, J. X., Li, P. Z. & Sun, Z. H. Research progress of estrogen in regulating gender differences in disease. *Life Sci. Res.* **28**, 189–214 (2024).

[49] Barrett, K. et al. Changes in the metagenome-encoded CAZymes of the rumen microbiome are linked to feed-induced reductions in methane emission from Holstein cows. *Front. Microbiol.* **1**, 1 (2022). 10.3389/fmicb.2022.855590 PMC9163818 35668758

[50] Karyani, T. Z., Ghattavi, S. & Homaei, A. Application of enzymes for targeted removal of biofilm and fouling from fouling-release surfaces in marine environments: A review. *Int. J. Biol. Macromol.* **253**, 127269 (2023). 37804893 10.1016/j.ijbiomac.2023.127269

[51] Tarracchini, C. et al. Genetic strategies for sex-biased persistence of gut microbes across human life. *Nat. Commun.* **14**, 4220 (2023). 37452041 10.1038/s41467-023-39931-2 PMC10349097

[52] Wang, L., Ravichandran, V., Yin, Y., Yin, J. & Zhang, Y. Natural products from mammalian gut microbiota. *Trends Biotechnol.* **37**, 492 (2019). 30392727 10.1016/j.tibtech.2018.10.003

[53] Cohen, Y. & Borenstein, E. The microbiome’s fiber degradation profile and its relationship with the host diet. *BMC Biol.* **20**, 266 (2022). 36464700 10.1186/s12915-022-01461-6 PMC9721016

[54] Minor, C. M. et al. A genomic analysis reveals the diversity of cellulosome displaying bacteria. *Front. Microbiol.* **15**, 1 (2024). 10.3389/fmicb.2024.1473396 PMC11557425 39539715

[55] Sonbol, H. S. & Jalal, R. S. Functional profiling of abundant glycosyltransferases in the rhizospheric bacteriome of Abutilon fruticosum. *Rhizosphere-Neth* **33**, 101001 (2025).

[56] Kadyan, S., Park, G., Wang, B. & Nagpal, R. Dietary fiber modulates gut microbiome and metabolome in a host sex-specific manner in a murine model of aging. *Front. Mol. Biosci.* **10**, 1 (2023). 10.3389/fmolb.2023.1182643 PMC10345844 37457834

[57] Mao, J. et al. High concentrate diets altered the structure and function of rumen microbiome in goats. *Front. Microbiol.* **15**, 1 (2024). 10.3389/fmicb.2024.1416883 PMC11322510 39144219

