# Supplementary material for: Metagenomic sequencing reveals the taxonomic and functional characteristics of rumen microorganisms in Dongliu buffalo
Source: Sci Rep. 2025 May 26;15:18398. doi: 10.1038/s41598-025-03059-8 (PMC12106671; doi:10.1038/s41598-025-03059-8)
Supplement: Supplementary file 1 — Supplementary Information 1. [file 41598_2025_3059_MOESM1_ESM.pdf]

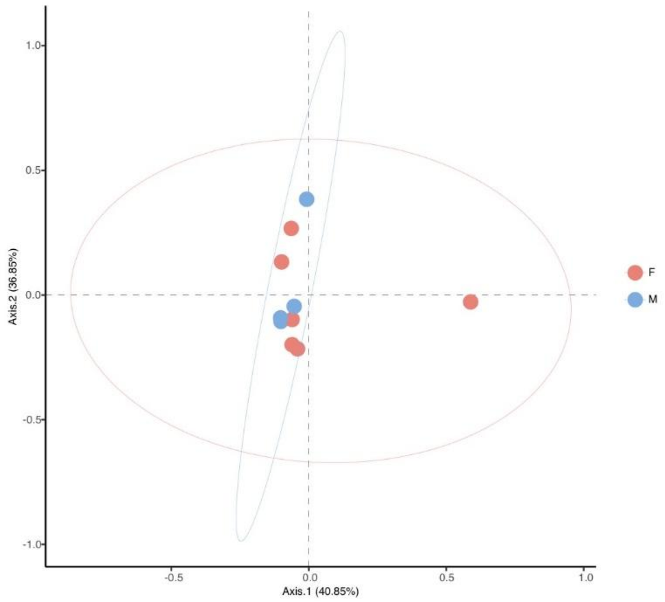

Figure S1 Principal component analysis (PCoA) based on unweighted UniFrac distance shows the distribution between samples of different groups

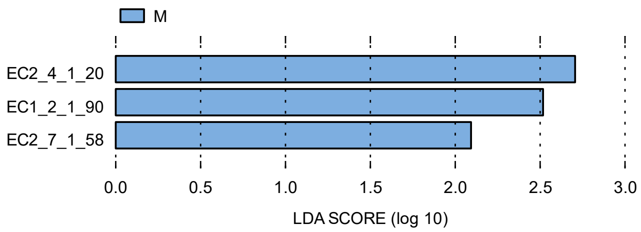

Figure S2 Linear discriminant analysis (LDA) effect size analysis (LEFSe) to identify carbohydrate met abolic pathway between sex groups.
